# Health in National Climate Change Adaptation Planning

**DOI:** 10.1016/j.aogh.2015.07.001

**Published:** 2015

**Authors:** Kristie L. Ebi, Elena Villalobos Prats

**Affiliations:** ∗ Department of Global Health, University of Washington, Seattle, WA; † Department of Public Health and Environment, World Health Organization, Geneva, Switzerland

## Introduction

Adaptation has been a critical component of the international negotiations under the United Nations Framework Convention on Climate Change (UNFCCC) from the beginning. When signing the convention, all countries agreed to produce regular national communications covering their emissions inventory, current impacts and projected risks of climate change, adaptation options to prepare for and reduce those risks, and mitigation options to reduce greenhouse gas emissions and transition to a green economy. The least developed countries (LDCs) also developed National Adaptation Programmes of Action (NAPAs) to identify their urgent and immediate adaptation needs. Building on the experience gained through the NAPA process, including implementation of adaptation options to address urgent national needs, National Adaptation Plans (NAPs) are under development by LDCs and other developing countries to identify and address medium and long-term adaptation needs.

The NAP process was initiated at the UNFCCC Conference of the Parties (COP) meeting in Cancun, Mexico, in 2010, at which countries acknowledged that national adaptation planning is an important process by which developing countries can assess their vulnerabilities, risks, and adaptation options.^1^ Further, the COP acknowledged that because the development challenges for LDCs are magnified by climate change, adaptation planning should move from primarily focusing on current climate variability and recent climate change to focusing more broadly on increasing resilience within the context of sustainable development planning. The COP established the NAP process to facilitate effective and efficient adaptation planning in developing countries. The UNFCCC Least Developed Country Expert Group (LEG) was mandated to develop technical guidelines to support the NAP process.^1^

The agreed objectives of the NAP process are to (1) reduce vulnerability to current effects and future climate change-related risks, and (2) facilitate the mainstreaming of climate change adaptation into development planning and other strategies within all relevant sectors and at local to national scales of governance. The COP also agreed that enhanced action on adaptation should do the following:

• Follow a country-driven, gender-sensitive, participatory, and fully transparent approach, taking into consideration vulnerable groups, communities, and ecosystems.
• Be based on and guided by the best available science and, as appropriate, traditional and indigenous knowledge, and employ gender-sensitive approaches with a view to integrating adaptation into relevant social, economic, and environmental policies and actions, where appropriate.
• Not be prescriptive, nor result in the duplication of efforts undertaken in a country, but rather facilitate country-owned, country-driven action.

Using the LEG technical guidelines for conducting a NAP, the World Health Organization (WHO) developed *Guidance to Protect Health From Climate Change Through Health Adaptation Planning*, taking into consideration the physical, social, and biological determinants of health.^2^ The goal of conducting an H-NAP is to strengthen health systems to protect health from climate variability and change; this includes addressing upstream drivers of health risks. The determinants of a climate-sensitive health outcome interact with development patterns and other factors to create baseline vulnerability to climate change. Future vulnerability will be partially determined by the effectiveness of policies and programs to prepare for, respond to, and recover from the consequences of changing weather patterns. That is, the success of development policies in a particular location will depend to some degree on climate change, and the magnitude and pattern of climate variability and change will depend on development policies.

The WHO guidance aims to ensure that the health and environment sector follows a systematic process to do the following:

• Engage in the overall NAP process at the national level.
• Identify national strategic goals for building health resilience to climate change.
• Develop a national plan with prioritized activities to achieve these goals, within a specific period and given available resources.

The health component of a NAP (H-NAP) follows the principles stated in the NAP guidance:

• Ensure that the NAP is a country-driven process owned by the respective countries.
• Ensure that health adaptation planning is based on the best available evidence. Any adaptation plan should aim to strengthen the development and availability of evidence, build the data and reduce knowledge gaps, and inform relevant policies.
• Build on existing national efforts on health adaptation to climate change, including assessments, and development and implementation of policies and programs at local to national levels.
• Integrate health adaptation to climate change into national health planning strategies, processes, and monitoring systems.
• Provide for a flexible and context-specific approach to health adaptation to climate change. National circumstances and available information and experience on health and climate variability and change will determine the scope, institutional arrangements, and resources required for proper implementation of the health component of the NAP.
• Maximize synergies across sectors, mainly across those that determine health, such as the food, water, energy, and housing sectors. This calls for developing relevant health indicators within the adaptation monitoring systems in these sectors, ensuring that health considerations are integrated into adaptation planning to avoid maladaptation.
• Ensure that the health adaption plan feeds into and coordinates with the overall national adaptation process.
• Pilot approaches that promote an iterative process for health adaptation to climate variability and change, producing plans over specified time horizons.
• Promote intercountry collaboration across agencies and stakeholders and harmonize adaptation approaches at subregional levels.

## H-NAP Process

**Figure 1:**
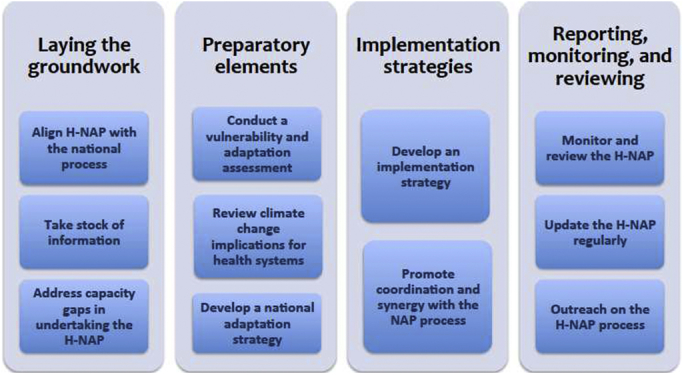
Elements and steps in conducting the health component of a national adaptation plan (H-NAP). (Based on WHO 2014.)

The elements of the H-NAP process are as follows: (1) laying the groundwork; (2) preparatory elements; (3) implementation strategies; and (4) reporting, monitoring, and reviewing (Fig. 1). These are broadly related to the phases of a project cycle (identification, formulation, implementation, and monitoring and evaluation). The WHO guidance provides a brief description of elements and steps, along with links to materials and tools to help support the process, and some examples from health adaptation projects.^2^

### Element 1: Lay the groundwork and Address Gaps in Undertaking the H-NAP Process

#### Aligning the health adaptation planning process with the national process for developing a NAP

Coordination between the health sector and the national adaptation planning process is necessary to do the following:

• Support integration of the health risks of climate variability and change into national adaptation plans and into national health planning processes.
• Promote implementation of health adaptation policies and programs at national to local levels.
• Coordinate the overall adaptation process.

The coordination between the Thailand Ministry of Health and the national adaptation planning led by the National Climate Change Committee is an example of this process. Furthermore, such coordination is critical to facilitate access to adaptation funds established by international donors and development partners to prepare for and manage the risks of climate variability and change. Health is underrepresented in these funds. A review of the inclusion of health within NAPAs concluded that 39 of 41 (95%) NAPAs identified health as a priority sector negatively affected by climate change, and 30 of 41 (73%) countries listed health interventions among their priority adaptation needs and proposed actions.^3^ However, only approximately 4% of the portfolio of the Least Developed Countries Fund supported health adaptation. Having health represented in the overall national adaptation planning process could facilitate access to urgently needed adaptation funds to address current and projected health risks of climate change. The Intergovernmental Panel on Climate Change concluded in 2014 that climate change has already contributed to levels of ill health and that major changes are projected across all emission pathways.^4^ Lack of adaptation funding to address these risks will affect current and future population health, the foundation of national development.

#### Taking stock of available information

This step is essentially a situational analysis of the weaknesses, opportunities, and threats of climate variability and change to human health; knowledge of factors that could increase or decrease vulnerability to these risks (eg, vaccination and access to improved sanitation); adaptation policies and programs under way to manage climate-sensitive health outcomes (eg, national malaria control programs); and national and regional knowledge and capacity gaps necessary to address in the H-NAP process.

A critical challenge in many low- and middle-income countries is the limited data on climate-sensitive health outcomes, particularly the number of cases of nonreportable infectious diseases such as dengue fever or diarrheal disease. Even when data are available, there may be limited capacity to analyze the data to determine the magnitude and pattern of associations between weather and/or climate and health outcomes. For example, a series of studies in resource-poor settings were conducted to enhance understanding of the mortality effects of weather conditions in parts of Africa and Asia.^5^ The INDEPTH network has been collecting high-quality standardized data on demographics and mortality for geographically defined populations over many years, resulting in high-quality registers representative of regions where such information is limited. For example, one of the studies focused on informal settlements in Nairobi, Kenya, and found significant associations between extreme hot and cold temperatures, increasing amounts of rainfall, and deaths in children from infectious and noncommunicable diseases.^6^ Across the 6 studies, children were the most vulnerable.^5^ A 2012 study not only described the observed trends in temperature and rainfall in the study regions but also projected substantial shifts in a few study areas.^7^ These results underscore the need for integrated surveillance to provide evidence of trends and the concern that the burden of climate-sensitive health outcomes could increase significantly over coming decades. Such analyses can inform health adaptation policies and decision making, strengthening health systems to reduce these risks. However, human and financial resources are presently insufficient to expand networks such as INDEPTH or to develop new networks to conduct monitoring and surveillance for climate-sensitive health outcomes.

Weather and climate information should also be identified in this step, including temperature data if heat-related morbidity and mortality are of concern and precipitation data for analyzing the associations between a wide range of infectious diseases and rainfall and flooding. This step can strengthen the collaborations between ministries of health, universities and other organizations, and national hydrometeorologic services and organizations. Early warning systems for climate-sensitive infectious diseases developed as part of a health adaptation project in the Kingdom of Bhutan are an example of a successful collaboration between the ministry of health and the national hydrometeorologic services.^8^ Another successful collaboration was established in China to effectively protect the health of vulnerable populations to heat stress (https://www.youtube.com/watch?v=QQoZUjEvNJE). Access to weather and climate data can be challenging in some countries, so promoting better collaborations early in the NAP process can be helpful.

#### Identify approaches to address capacity gaps and weaknesses in undertaking the H-NAP process

The analyses conducted in the previous step will indicate whether additional efforts are needed to undertake the H-NAP process, such as identifying where augmentation of current data collection is needed to provide adequate evidence or where training is needed to conduct data analyses. Countries are just beginning to develop their H-NAPs, so it is expected that lessons learned and best practices in addressing capacity gaps will emerge over the next few years.

### Element 2: H-NAP Preparatory Elements

#### Conduct a health vulnerability and adaptation assessment, including short- to long-term adaptation needs in the context of development priorities

WHO, the European Centre for Disease Prevention and Control (ECDC), and others have published guidance on conducting a health vulnerability and adaptation assessment.^9^, ^10^ Many countries have conducted some form of a vulnerability and adaptation assessment, although few are comprehensive. An example of a comprehensive assessment is one conducted for Kiribati, a low-lying, resource-poor Pacific atoll nation.^11^ The process assessed risks to health and sources of vulnerability. The high-priority adaptation needs identified included policies and programs to improve management of water safety and water-borne disease; food safety and food-borne diseases; and vector-borne diseases. Improving disease surveillance was also critical to managing most climate-related health risks in Kiribati.

Assessment of the risks of climate change is an iterative process rather than a one-off activity.^12^, ^13^ Vulnerabilities are likely to shift as climate and development proceed, technologies advance, and new evidence and knowledge emerges on trends, projections, and best practices in adaptation. Therefore, conducting regular vulnerability and adaptation assessments that can then be incorporated into national adaptation plans is a recommendation in the H-NAP process.

#### Review implications of climate change on health-related development goals, legislation, strategies, policies, and plans

Strengthening health systems is the first area of focus for most national health adaptation plans to reduce the current burden of climate-sensitive health outcomes. Components of strengthening health systems include the essential public health services. Of particular importance are the following elements^14^, ^15^:

• Building awareness of the health risks of climate change and the adaptation and mitigation actions needed to protect health today and in the future.
• Building the capacity of public health and health care professionals to monitor, diagnose, and treat cases of climate-sensitive health outcomes, even when they change their incidence, seasonality, and geographic range.
• Developing new methods and tools for preparing for, coping with, and recovering from outbreaks of climate-sensitive diseases, such as early warning systems based on environmental information.
• Strengthening monitoring and surveillance programs to track climate-sensitive health outcomes and improving health services delivery to ensure prompt and effective treatment.
• Strengthening evaluation (and learning) programs designed to identify lessons learned and best practices in health adaptation and how these can be scaled up and out within and across countries.
• Improving governance and policies for managing the health risks of climate variability and change.
• Conducting research to develop new insights and innovative solutions to, for example, emerging and re-emerging infectious diseases.
• Raising the necessary human and financial resources to undertake these actions.

Building the resilience of current and planned health programs includes strengthening coordination across departments in ministries of health mandated to manage climate-sensitive health outcomes (including vector-borne diseases, diarrheal diseases, food and water safety, noncommunicable diseases, and nutrition), and across ministries and departments whose policies and programs can affect these health outcomes. Coordination with sectors such as agriculture, water, and disaster risk management will facilitate identifying potential synergies and promote health co-benefits (see the example of Indonesia in a later section). For example, choices made on enhancing crop yields through irrigation or microdams could not only reduce food insecurity but could also provide breeding grounds for mosquitoes, snails, and other disease vectors.

#### Develop a national health adaptation strategy that identifies priority adaptation plans

The H-NAP should specify the priority strategies designed to address the adverse health risks of climate variability and change and include a detailed plan of action. Having a comprehensive vulnerability and adaptation assessment will facilitate developing strategies and implementation plans. Using a theory-of-change approach (eg, a process for defining how to achieve a long-term goal by identifying necessary early and immediate changes) can facilitate identification of priority strategies. Conducting a theory-of-change process includes working with stakeholders to identify the long-term change being sought; the process and sequence of change, including the socioeconomic and political conditions and the actors able to influence them; explicit statements of the assumptions about how change might happen; and a diagram and narrative summary of the process and expected outcome. The overarching strategy is generally to increase the resilience of health systems to the health risks of climate variability and change; this includes ensuring that health systems can prepare for, respond to, cope with, and recover from climate-related effects.

The H-NAP should include detailed institutional arrangements to implement and engage relevant stakeholders; immediate and medium- and long-term health adaptation goals, with a time frame for implementation; a monitoring and evaluation framework; and approaches to ensure adequate human and financial resources are available to implement the plan.

For example, African ministers of health and of environment have begun jointly implementing the framework for Public Health Adaptation to Climate Change.^15^ The guiding principles are evidence-based planning, country ownership and community participation, intersectoral cooperation and collaboration, synergies with other public health initiatives, and advocacy at national and international levels. Implementation requires multisectoral Country Coordination Committees (CCCs), which are national technical and advisory entities intended to address health and environment issues—including climate change—that should include government representatives, development partners, and civil society. The proposed template for the adaptation plan includes risk and capacity assessment; capacity-building; integrated environment and health surveillance; response; research; monitoring and evaluation; and coordination and management. Following this process will put countries on pathways to become more resilient to the health risks of climate change.

### Element 3: Implementation Strategies

#### Develop an implementation strategy for operationalizing the H-NAP and integrating climate change adaptation into health-related planning and processes at all levels, including enhancing the capacity for conducting future H-NAPs

This step calls for identifying approaches to ensure that the health risks of climate variability and change are considered in health planning at the local and the national scale.

Differences between an implementation strategy for the health risks of climate change and an implementation strategy for other health risks include a strong focus on integrating health systems policies and programs at local to national scales, where needed; consideration of much longer time frames for implementation; embedding implementation into an explicit iterative and adaptive management framework; and coordinating and collaborating across sectors.^12^, ^13^

The longer time frame for implementation includes considering a range of risks that are projected to change with changing weather patterns. For example, if projections suggest that vector-borne diseases could change their geographic range, then planning is required to determine where and when surveillance should be expanded. Another example is evaluating whether critical health care infrastructure could be at risk from rising sea levels or increasing storm surges in the coming decades and developing a strategy for moving or replacing those at high risk.

The effectiveness of health systems strategies and policies are likely to change as the climate continues to change and as development choices affect vulnerability and the ability to cope with the health risks of climate variability and change. Therefore, strategies and policies should be designed and implemented within an adaptive management framework.^12^, ^13^ This includes regularly evaluating whether policies and programs continue to be effective or whether adjustments are needed. Adaptive management also calls for explicit exploration of different approaches to achieve objectives (eg, reducing vulnerability to particular health outcomes), testing alternatives to determine which are more effective, and then using the lessons learned to update knowledge and make modifications to policies and programs. This exploration is helpful when choosing which programs to scale out to wider geographic regions.

#### Promote coordination and synergy with the NAP process, particularly with sectors that can affect health, and with multilateral environment agreements

This step calls for including a description in the H-NAP of how it will promote the national strategies identified in the overall NAP process and how health adaptation will coordinate with other sectors in promoting human health and well-being.

Because many of the upstream drivers of the health risks of climate change arise from outside the sector, multisectoral approaches are required for effective adaptation.^16^ Closer partnerships across sectors and, ultimately, better governance can arise from understanding the different institutions and organizations whose decisions can affect population health. Partnering mechanisms, at local to national scales, should include sectoral collaboration, policy coordination, and increasing coherence of risk management approaches. One way to facilitate cross-sectoral coordination is to include health indicators within the national indicator framework (see the next section).

Bowen et al. proposed a multilayered governance network for identifying the actual decision-making actors, their roles, and their level of influence in health adaptation options.^17^ The network includes 4 elements: social capital, non–state-based actors, informal networks, and bridging organizations. These elements can be explicitly incorporated into implementation strategies to facilitate effective and efficient adaptation through strengthening partnerships and collaboration mechanisms.

Social capital includes the capacity of a population or community to work together as a self-organizing unit to achieve particular goals.^18^ The activities undertaken can, depending on the circumstances, facilitate adaptation. Social capital is also an important driver of some health outcomes (eg, mental health).^19^ Greater efforts are needed to identify the enabling conditions for norms and behaviors that will support greater awareness of and actions to reduce the risks of climate change.

It also is important to link with multilateral environmental agreements to identify opportunities for synergies across adaptation and mitigation efforts. For example, greening of the health care sector can promote adaptation, by identifying modifications to increase the resilience of facilities and infrastructure to extreme weather and climate events. Greening also can promote mitigation by identifying energy efficiency opportunities in energy intensive facilities such as hospitals.

If the H-NAP is structured from the beginning to integrate with the NAP process, then this step can identify where, for example, formal memorandums of understanding or other mechanisms can institutionalize collaborations developed during the H-NAP. An example is the Republic of Indonesia climate change sectoral roadmap (ICCSR) developed in 2009 to provide input for the 5-year medium-term development plan (RPJM) 2010-2014 and for subsequent RPJMs until 2030, placing particular emphasis on the challenges emerging in 9 particular sectors, including health.^20^ Each sector developed a roadmap that included a policy analysis, formulation of a vision on climate change, and identification of a pathway of actions to accomplish the vision. The national development planning agency is addressing challenges and opportunities across sectors through coordination of the work of all ministries, departments, and agencies. The roadmap includes prioritized recommendations that reflect the linkages across policies, programs, and plans for different sectors and donor programs/projects.

### Element 4: Reporting, Monitoring, and Review

#### Monitor and review the H-NAP to assess progress, effectiveness, and gaps

An objective of the H-NAP process is to strengthen national systems for monitoring and evaluating the burden of climate-sensitive health outcomes and to align those systems with international reporting.^21^ Guidance on what needs to be included in a national health information system should be developed during the H-NAP process, taking into consideration the priority health outcomes of concern over the coming decades, the human and financial resources needed to augment surveillance programs, whether additional capacity needs to be built within the ministry of health and other organizations for monitoring and evaluation of climate-sensitive health risks, and the recommended indicators for monitoring the process of adaptation and the effectiveness of the products of specific adaptation programs (eg, early warning systems).

Indicators for monitoring, evaluation, and learning can include the following:

• The number and geographic distribution of cases and deaths (and trends over time) in climate-sensitive health outcomes. Health outcome data should be at least disaggregated by age and gender to identify high-risk population subgroups and to facilitate design of tailored interventions. Other variables to consider when developing indicators depend on the health outcome of interest; for example, socioeconomic variables where income distribution is important or the prevalence of chronic diseases.
• Trends in factors that increase or decrease vulnerability and exposure to the hazards associated with changing weather patterns and sea level rise, such as urbanization, access to improved water sources, and social capital.
• Weather and climate variables, such as average and extreme temperature and precipitation, trends in the frequency and intensity of extreme weather and climate events, and sea level rise. Other environmental variables also may be useful, such as measures of soil moisture or stream flow.
• The effectiveness of adaptation policies and programs, such as whether a particular option decreased the number of people at risk during a flood or increased the capacity of health care professionals to use weather and climate variables to forecast health risks.
• The process of adaptation, including tracking the progress on identifying and scaling out lessons learned and best practices.

Because the health sector has been managing the risks of climate-sensitive health outcomes for decades to centuries, there are well-established indicators for monitoring the burden of these outcomes, such as the number of children who are undernourished, the number of deaths from specific vector-borne diseases, or the proportion of a community or region with access to improved drinking water sources and sanitation.^22^ In some cases, it would be helpful to increase the frequency of weekly or monthly monitoring because developing robust early warning systems generally requires daily data so that any associations between weather or climate and health variables are discernible. Where changes in the geographic range of vector-borne diseases are of concern, surveillance may need to be expanded into new regions. Working with stakeholders across local to national scales during the H-NAP will facilitate identifying where gathering additional health information would promote resilience.

Quantitative and qualitative indicators can be used to monitor trends in factors that can affect vulnerability. Indicators of important social determinants of vulnerability or exposure can include the extent to which gender is a key driver of vulnerability, metrics of the strength of social capital for promoting health adaptation or coping with extreme weather and climate events, and the effectiveness of bridging organizations in reducing vulnerability.

The H-NAP will identify the weather and climate variables associated with adverse health outcomes. These variables are generally measured by national hydrometeorologic services but may not be measured in locations important for health adaptation. Collaborating with these services will help ensure that information is available on these variables for monitoring the effectiveness of adaptation interventions.

Although monitoring and evaluation of the effectiveness of adaptation options are well established in health systems, current approaches were not designed for issues such as climate change where, for example, the magnitude of the “exposure” to weather and climate events cannot be reduced until at least mid-century; where local socioeconomic and environmental conditions are key determinants of the magnitude and pattern of risks; and where upstream drivers mainly arise from sectors outside health. Monitoring and evaluation frameworks also need to be adjusted to describe explicit baselines against which the effectiveness of adaptation options can be measured over time and to inform future modification of adaptation options. Baseline variables include those describing the patterns of vulnerability, patterns of weather and climate variables important for health, the effectiveness of current health systems interventions, and the capacity of health systems to manage the health risks of climate variability and change. Low- and middle-income countries are developing baselines as they implement adaptation projects (cf http://www.who.int/globalchange/projects/en/).

WHO is developing summary climate change and health country profiles to provide a baseline for monitoring future progress. The profiles will include information on current weather and climate hazards and health impacts; current burdens of climate-sensitive health outcomes; future exposures and health risks under different climate change scenarios; opportunities for health gains through climate change mitigation and adaptation; and policy information related to health and climate change.

When monitoring effectiveness, it is important to capture not only best practices but also barriers and constraints and how they were overcome (if they were). Scaling out adaptation options to other regions will be easier and more effective when there is a more complete understanding of the process of adaptation.

Having an adaptation strategy does not guarantee that it is implemented, and implementing a strategy may not be as effective as possible without including other sectors and nonstate actors. It is therefore important to include indicators focused on the process of adaptation and the extent to which adaptation interventions are effective. To start, annual, mid-term, and final review of the implementation of the H-NAP and of recommended adaptation programs should be considered in the list of indictors. Indicators monitoring interactions across sectors could include, for example, the frequency of intersectoral meetings and whether memoranda of understanding are actually facilitating data sharing.

The monitoring, evaluating, and learning framework should include milestones and should be designed for regular evaluation to introduce modifications when needed.

#### Update the health component of the NAP in an iterative manner

The magnitude and pattern of the health risks of climate variability and change will shift as climate and development proceed, which will require revisiting the H-NAP to ensure continued relevance and effectiveness. Revisions to the H-NAP should also take into account experience gained with implementing adaptation options, knowledge gained from research, and changes in institutional structures. The updating process should be flexible, revisiting the H-NAP whenever major changes occur that could alter the previous conclusions and approaches to adaptation. An update should also be considered when the national adaptation plan is revised.

#### Outreach on the H-NAP process, including reporting on progress and effectiveness

Outreach is invaluable for increasing the preparedness of communities, health services providers, and other key stakeholders. A communication strategy should be developed from the beginning of the H-NAP process.^9^ Stakeholders should include management units within the ministry of health responsible for climate-sensitive health outcomes, representatives from health care providers, representatives from other sectors, community representatives, and others who could be affected by or who could affect the success of the adaptation plan.

The NAP process will define report requirements and the timeframe for doing so. To the extent possible, health data generation, compilation, analysis, synthesis, communication, and use for decision-making should be aligned with these requirements so that the H-NAP can ensure the best possible information is included in the national adaptation plan, highlighting the potential health risks of climate variability and change, and the opportunities for proactively managing these risks to reduce the current and projected burden of climate-sensitive health outcomes.

## Discussion

All actors in health systems, from research scientists to ministries of health staff to health care providers, are responsible for understanding, engaging in, and supporting the health component of a national adaptation plan. Most H-NAP elements and steps will be familiar; understanding and managing risks are at the core of health systems. However, climate variability and change bring new dimensions that will require training and capacity building to mainstream climate change concerns into health system strategies, policies, and programs and into research and development. Focusing on strengthening health protection systems for climate-sensitive health outcomes is important, including increasing access to vaccination, safe water, and improved sanitation. But that is only the start. Risks will evolve with climate and development, requiring proactive planning, implementation, and monitoring and evaluation if health systems are to be prepared to reduce risks to the fullest extent possible.

There also are key research gaps that need to be filled, particularly in low-income countries.^4^ National governments and international research funders can help fill those gaps, supporting analysis of existing data sets, including projecting how health risks could change under different possible futures, and supporting the collection of additional longitudinal data where gaps exist. This research should include an explicit focus on upstream drivers of climate-sensitive health risks. This critical research's limited funding constrains the design and implementation of effective and efficient adaptation options. More methods and tools are coming online, such as early warning systems that can effectively increase resilience to some negative health outcomes. Additional efforts are required to increase their uptake and effective use.

The health risks of climate variability and change are already unacceptable, with the burden projected to increase as the climate continues to change. Proactive and efficient adaptation can reduce health burdens over coming decades but cannot eliminate all risks.^4^ Mitigation is equally as important as adaptation; rapid and substantial reductions in greenhouse gas emissions are required to reduce risks past mid-century. But even with substantial reductions and with significant investments in adaptation, climate-sensitive health risks in excess of what countries will be able to manage will be a feature of coming decades. Conducting an H-NAP is the best opportunity for preparing for very different socioeconomic and environmental conditions that will likely challenge the ability of health systems to maintain and improve population health.
